# Cultural change demands proportionate societal response in the handling of suspected FGM/C cases

**DOI:** 10.1038/s41443-022-00535-x

**Published:** 2022-02-07

**Authors:** Sara Johnsdotter, Lotta Wendel

**Affiliations:** Faculty of Health and Society, Malmö University, Malmö, Sweden

**Keywords:** Quality of life, Disease prevention

We read with great interest the article by Karlsen et al. with the title “Available evidence suggests that prevalence and risk of Female Genital Cutting/Mutilation in the UK is much lower than widely presumed—policies based on exaggerated estimates are harmful to girls and women from affected communities,” [1] and would like to affirm that their findings have relevance beyond the situation in the UK.

An overview of criminal FGM/C court cases in Europe demonstrates the scarcity of confirmed illegal FGM/C cases among affected immigrant communities during several decades [2]. Although such a survey cannot account for unreported cases of illegal FGM/C, the low number of cases taken to court gives important clues to the current situation. When such data are triangulated with other register data, as in the study by Karlsen et al., the conclusion is corroborated: the expectations of secretive FGM/C activities reflected in overestimated risk and prevalence figures are apparently inaccurate.

In our ongoing research project in Sweden, we currently have access to 170 FGM/C-related police documents: reports to the police about suspected performed or pending FGM/C. A specific criminal law provision banning FGM/C was introduced in Sweden in 1982, and the first criminal investigation regarding FGM/C took place in 1996. Some 130 of those 170 reports resulted in formal criminal investigations. Yet only three cases have led to prosecution (all of them ended in prison sentences): two cases in 2006, in which FGM/C was said to have been performed in Somalia, and one case in 2018 when a father was sentenced to 6 months in prison for stating his wish to have his two daughters circumcised in Nigeria in the future. As regards the rest of the suspected cases in the Police register, only five girls were born or grew up in Sweden. In those cases, the FGM/C was performed in African countries by people who were not indictable in the Swedish court system. In the preponderance of cases, no FGM/C could be found through medical examination, or the investigation indicated that the procedure had been performed prior to migration.

Like Karlsen et al. we are concerned about inflated estimates of girls at risk of FGM/C and notions about illegal underground activities, assumptions that are abounding in the public discussion in most European countries. Often it is claimed that the lack of convictions is due to ignorance or negligence on the part of the authorities, or that legislation must be strengthened in order to intercept the illegal cases. The UK is a prime example of this, now having the harshest laws and most repressive policies to tackle FGM/C in Europe. Yet, as Karlsen et al. show regarding the UK, harsh policies and surveillance do not seem to result in a higher number of identified illegal cases. Data from Scotland are illustrative: out of 52 referrals or child welfare concerns between April 2013 and September 2016, the investigations revealed that in no case had FGM/C been performed [3].

Consequently, there is good reason to consider the possibility that large-scale cultural change regarding practices and views of FGM/C has taken place among many affected immigrant communities in European host countries [4]. Instead of expecting “culture” to be a static phenomenon and supposing that traditions always are “deeply rooted,” one ought to focus on the dynamic aspects of lived culture. Migration seems to foster new strategies in new contexts, and in the case of FGM/C, many women who themselves have suffered from these practices now have the opportunity to escape them and better protect their own daughters [5].

The overblown risk and prevalence estimates that policymakers and the mass media offer thus seem to be seriously flawed, but they still underpin the authority measures of policing these communities. The relation between authority efforts to identify cases for prosecution and the scarcity of confirmed illegal cases demonstrates the importance of the principle of proportionality.

This principle is an established part of human rights and aims at insuring everyone to be treated by public authorities in accordance with the rule of law. An authority must not use more intrusive measures than are required with regard to the purpose. Where there is a choice between several appropriate measures, authorities must aim for the least onerous one. An action may be taken against a person only if the reasons for the action outweigh the inconvenience that the action entails for the person affected. Policies and practices can be justified only if they serve a legitimate aim and there is a reasonable proportionality between the means employed and the aim to be achieved.

When discussing what constitutes legitimate aims and proportional means, the principle of proportionality highlights the importance of human rights. The European Convention on Human Rights protects the right to privacy and to family life, and the right to physical integrity. These rights are incorporated into domestic British law by the Human Rights Act of 1998.

The interest of safeguarding human rights may often lead to contradictory considerations. The right to privacy and family life protects families from intrusions, while it also protects individuals from enforced traditional practices. The right to physical integrity entails a right for girls and women not to be cut, but it also provides protection for girls and women against forced genital examinations by public authorities.

The process when different legitimate values are weighed against each other involves a large measure of discretion. The outcome of the deliberation depends on how different interests and societal problems are articulated and constructed in the surrounding cultural and institutional context. This is what makes the article by Karlsen et al. so important. There is a clear need for factual information regarding the possibility of large-scale cultural change of practices and views of FGM/C and its consequences. Rule of law can be achieved only if the assessment by the authorities in individual cases is based on facts regarding the practice of FGM/C and not on exaggerated estimates and speculations.

A reasonable approach from authorities would be one characterized by balance: a position in which the police, prosecution, and child protection services strive to protect vulnerable girls and to safeguard their legal rights, while they do not condescend to exaggerations and hypervigilance. A genuine protection of young girls in the affected communities means not to expose them to discrimination and traumatic events in cases when there is low probability that illegal actions have taken place.
